# circAKT3 positively regulates osteogenic differentiation of human dental pulp stromal cells via miR-206/CX43 axis

**DOI:** 10.1186/s13287-020-02058-y

**Published:** 2020-12-09

**Authors:** Bo Zhang, Sibei Huo, Xiao Cen, Xuefeng Pan, Xinqi Huang, Zhihe Zhao

**Affiliations:** 1 State Key Laboratory of Oral Diseases & National Clinical Research Center for Oral Diseases, West China Hospital of Stomatology, Sichuan University, Chengdu, People’s Republic of China; 2 Department of Orthodontics, West China Hospital of Stomatology, Sichuan University, No. 14, 3rd Section, South Renmin Road, Chengdu, 610041 Sichuan People’s Republic of China; 3 Department of Stomatology, Children’s Hospital of Chongqing Medical University, Ministry of Education Key Laboratory of Child Development and Disorders, China International Science and Technology Cooperation Base of Child Development and Critical Disorders, Chongqing Key Laboratory of Pediatrics, National Clinical Research Center for Child Health and Disorders (Chongqing), Chongqing, People’s Republic of China; 4 Department of Temporomandibular Joint, West China Hospital of Stomatology, Sichuan University, Chengdu, People’s Republic of China

**Keywords:** Circular RNA, circAKT3, Human dental pulp stromal cells, Osteogenesis, MicroRNA, miR-206

## Abstract

**Background:**

Human dental pulp stromal cells (hDPSCs) are promising sources of mesenchymal stem cells (MSCs) for bone tissue regeneration. Circular RNAs (circRNAs) have been demonstrated to play critical roles in stem cell osteogenic differentiation. Herein, we aimed to investigate the role of circAKT3 during osteogenesis of hDPSCs and the underlying mechanisms of its function.

**Methods:**

We performed circRNA sequencing to investigate the expression profiles of circular RNAs during osteogenesis of hDPSCs. Quantitative reverse transcription polymerase chain reaction (qRT-PCR) was performed to detect the expression pattern of circAKT3 and miR-206 in hDPSCs during osteogenesis. We knocked down circAKT3 and interfered the expression of miR-206 to verify their regulatory role in hDPSC osteogenesis. We detected hDPSCs mineralization by alkaline phosphatase (ALP) and Alizarin Red S (ARS) staining and used dual-luciferase reporter assay to validate the direct binding between circAKT3 and miR-206. To investigate in vivo mineralization, we performed subcutaneous transplantation in nude mice and used hematoxylin and eosin, Masson’s trichrome, and immunohistochemistry staining.

**Results:**

Totally, 86 circRNAs were differentially expressed during hDPSC osteogenesis, in which 29 were downregulated while 57 were upregulated. circAKT3 was upregulated while miR-206 was downregulated during hDPSC osteogenesis. Knockdown of circAKT3 inhibited ALP/ARS staining and expression levels of osteogenic genes. circAKT3 directly interacted with miR-206, and the latter one suppressed osteogenesis of hDPSCs. Silencing miR-206 partially reversed the inhibitory effect of circAKT3 knockdown on osteogenesis. Connexin 43 (CX43), which positively regulates osteogenesis of stem cells, was predicted as a target of miR-206, and overexpression or knockdown of miR-206 could correspondingly decrease and increase the expression of CX43. In vivo study showed knockdown of circAKT3 suppressed the formation of mineralized nodules and expression of osteogenic proteins.

**Conclusion:**

During osteogenesis of hDPSCs, circAKT3 could function as a positive regulator by directly sponging miR-206 and arresting the inhibitive effect of miR-206 on CX43 expression.

**Supplementary information:**

The online version contains supplementary material available at 10.1186/s13287-020-02058-y.

## Background

Bone tissue is the critical supportive structure in craniofacial physiology. Numerous conditions, such as trauma, tumors, and necrosis, can cause bone defects [1]. Craniofacial structures are closely connected in functions, so bone defects are possible to ultimately result in extensive malfunction. Therefore, researchers are eager to figure out strategies to promote bone healing and reconstruct bone defects to avoid further damage in craniofacial region.

Mesenchymal stromal cells (MSCs) have attracted great attention as seed cells in osseous engineering to repair craniofacial bone defects [2]. A promising source of MSCs in bone tissue engineering is dental pulp tissue [3]. Initially discovered by Gronthos et al. [4], human dental pulp stromal cells (hDPSCs) are characterized by high levels of self-renewal and proliferation and multi-lineage differentiation capability [5]. They manifest similar clonogenic and proliferation properties to human bone marrow mesenchymal stromal cells (BMSCs) [4]. However, they can be more easily and non-invasively gained from removed teeth in comparison with BMSCs. Accumulating evidence has proved the potential of DPSCs in generating bone-like tissues and repairing bone loss [6–8]. For all these reasons, hDPSCs represent rising candidates for therapies of bone tissue repair.

The differentiation of MSCs is precisely modulated by a complex signaling network, in which non-coding RNAs (ncRNAs) are emerging as a novel group of active components [9]. As a newly discovered class of functional ncRNAs, circular RNAs (circRNAs) and their functional implications have ignited great interest in the field of bone regeneration. Though firstly thought to be a group of products of erroneous splicing [10], circRNAs have recently been proved abundant in human transcriptome and critical in cell functions and human diseases [11–16]. Circular RNAs are generated by the back-splicing process, and they are covalently closed loops, keeping them highly stable to RNase R digestion [17]. They can participate in the regulation of cell biological behaviors through multiple mechanisms, including sponging miRNA, interacting with RNA-binding proteins (RBPs), directly translating into proteins, and affecting U1 small nuclear ribonucleoproteins and polymerase II machinery to regulate the parental gene expression. Emerging studies have highlighted the potential functions of circRNAs in the osteogenesis of stem cells derived from various tissues [18]. However, the roles of circRNAs during the osteogenesis of hDPSCs are still ill-defined.

In this research, we aimed to identify key circRNAs that regulate osteogenic differentiation of hDPSCs and further investigate the underlying mechanism of their modulatory role to provide new insights for stem cell-based bone regeneration.

## Materials and methods

### Cell isolation, culture, osteogenic induction, and cell transfection

After getting informed consent from all participants and parents/legally authorized representatives of minors, we isolated hDPSCs from premolars and third molars of healthy donors (aged 14 to 25 years) and cultured them as described previously [19]. Briefly, we cultured hDPSCs in Dulbecco’s modified Eagle’s medium (HyClone, GE Healthcare) with supplemented with 10% fetal bovine serum (Gibco; Life Technologies) and 1% penicillin-streptomycin at 37 °C and 5% CO_2_. We used cells from passages 3 to 5 for experiments. We began osteogenic induction when hDPSC culture reached 80% confluence. We added 10 nM dexamethasone, 10 mM β-glycerophosphate, and 50 μg/mL vitamin C (Sigma-Aldrich) into basic medium for the osteogenic-induction medium (OM).

To knockdown circAKT3, sh1-circAKT3 or sh2-circAKT3 was cloned into pHBLV-U6-MCS-CMV-ZsGreen-PGK-PURO vector, and lentivirus was constructed by Hanbio Co., Ltd. (Shanghai, China). The sequences were provided in Table S1. We transfected microRNA (miRNA) mimics or miRNA inhibitor (Genepharma, Shanghai, China) with the help of Lipofectamine 3000 (Invitrogen, USA) to alter the expression of miR-206 (Table S1).

### circRNA sequencing

We collected hDPSCs cultured in OM for 0D and 14D for circRNA sequencing. We extracted the total RNAs using the TRIzol reagent (Invitrogen), examined the purity and concentration of RNAs by NanoDrop ND-1000 (NanoDrop Thermo), and detected the RNA integrity of samples by denaturing agarose gel electrophoresis. circRNAs sequencing and RNAs library construction were completed by CloudSeq Biotech Inc. (Shanghai, China) as described previously [20]. Briefly, the RNAs libraries were constructed by the rRNAs-depleted RNAs with TruSeq Stranded Total RNAs Library Prep Kit (Illumina, San Diego, CA, USA). The library quality was measured by BioAnalyzer 2100 system (Agilent Technologies, Inc., Richardson, TX, USA). The paired-end reads were acquired using the Illumina HiSeq 4000 sequencer. We applied threshold values of |Log2FC| > 1 and *P* value of 0.05 to select the differentially expressed genes.

We then used Gene Ontology (GO) and Kyoto Encyclopedia of Genes and Genomes (KEGG) pathway analyses to predict the functions of differentially expressed circRNA-associated genes. GO analysis included biological processes (BP), cellular components (CC), and molecular functions (MF). KEGG pathway analysis was applied to identify pathways related to the target mRNAs of differentially expressed circRNAs.

### Alkaline phosphatase (ALP) and Alizarin Red S (ARS) staining

We performed ALP staining with the BCIP/NBT Alkaline Phosphatase Color Development Kit (Beyotime Biotechnology, Shanghai, China). After 7 days of osteogenic induction, we fixed cells with 4% paraformaldehyde for 15 min in room temperature and performed ALP staining following the manufacturer’s instructions. We used Alkaline Phosphatase Assay Kit (Beyotime Biotechnology, Shanghai, China) to quantify of ALP activity. Cells were lysed and transferred to a 96-well plate. Reaction buffer and p-nitrophenol were then added following the kit instruction. The reaction system were incubated at 37 °C for 10 min and values of absorbance were measured at 405 nm by a Varioskan LUX microplate reader (Thermo Fisher Scientific).

After 14 days of osteogenic differentiation, we conducted ARS staining to detect the mineralized nodules with Alizarin Red S Stain Solution (Cyagen, China) according to the manufacturer’s instructions. Briefly, we fixed the cells with 4% formaldehyde for 15 min and stained them with 0.1% ARS (pH = 4.2) at 37 °C for 20 min. Then, we removed the staining solution and rinsed cells 3 times with deionized water. To quantify ARS staining, we solubilized the samples by 10% acetic acid according to the kit’s instructions (ScienCell Research Laboratories), and then the solution was added in a 96-well plate to read the absorbance at 405 nm.

### Quantitative reverse transcription polymerase chain reaction (qRT-PCR)

We extracted total RNA from hDPSCs using TRIzol (Invitrogen, USA) and synthesized cDNA from 1 μg total RNA with reverse transcriptase (TaKaRa Biotechnology, Otsu, Japan). Random primers were used to analyze circRNAs. qRT-PCR was performed using SYBR-Green PCR Master Mix Kit (Takara, Dalian, China) for PCR reactions. Gene expression level was normalized to glyceraldehyde 3-phosphate dehydrogenase (GAPDH) or U6. Table S2 listed the primer sequences used for circAKT3, miR-206, connexin 43 (CX43), ALP, runt-related transcription factor 2 (RUNX2), osteocalcin (OCN), U6 (control for miRNAs), and GAPDH (control for mRNAs and circRNAs). The results were calculated following the ΔΔCT method and presented as fold changes relative to GAPDH or U6.

### Dual-luciferase reporter assay

0.16 μg circAKT3 luciferase reporter plasmids were transfected into HEK 293 T cells (from National Collection of Authenticated Cell Cultures) with 5 pmol miR-NC or miR-206 mimic using LipoFiter (Hanbio, Shanghai, China). After transfection for 48 h, the luciferase activities were measured with the Dual-Luciferase Reporter Assay System (Promega, Beijing, China). Relative luciferase activity was normalized to Renilla luciferase activity.

### Western blot

We extracted the total cell protein from cultured cells using radioimmunoprecipitation assay (RIPA) lysis buffer and determining protein concentration by the BCA Protein Assay Kit (Thermo). Equal quantities of protein samples were separated by 12% sodium dodecyl sulfate-polyacrylamide gel electrophoresis (SDS-PAGE) and transferred onto polyvinylidene difluoride (PVDF) membranes (Millipore, Billerica, MA, USA). We then incubated PVDF membranes with primary antibodies.

against RUNX2 (1:1000, Abcam), OCN (1:1000, Abcam), CX 43 (1:1000, Proteintech), ALP (1:1000, Huabio), and GAPDH (1:1000, Abcam) overnight at 4 °C. After washed with TBST three times, PVDF membranes were incubated with corresponding secondary antibodies (1:5000, Abcam) for 2 h. The band intensity was measured by ImageJ software. The signal of all the target bands was normalized to GAPDH band.

### In vivo bone formation of HDPCs

We performed in vivo bone formation analysis using subcutaneous transplantation as described previously [21]. In brief, we soaked beta-tricalcium phosphate (β-TCP) blocks (5 mm × 5 mm × 2 mm) in OM for 30 min at 37 °C. Then we dripped a suspension of 40 μL OM containing 1 × 10^6^ hDPSCs on every β-TCP block and incubated them for 24 h at 37 °C with a 5% CO_2_ incubator. We anesthetized 6-week-old BALB/c immunodeficient nude mice (*n* = 4) by isoflurane inhalation and created subcutaneous dorsal pockets. The scaffolds of the sh-NC group and sh1-circAKT3 group were carefully transplanted into the left and right dorsal subcutaneous region respectively. We collected the β-TCP/hDPCs composites after 2 months, fixed them with 10% formalin for 48 h, and decalcified them with 10% ethylenediaminetetraacetic acid (EDTA; pH = 7.4) for 1 month.

### Hematoxylin and eosin (H&E) staining, Masson’s trichrome, and immunohistochemistry (IHC) staining

The specimens were washed, dehydrated, and embedded in paraffin after decalcification and then sectioned at 5 μm thickness. Masson’s trichrome staining was performed following the instruction of Masson’s Trichrome Stain Kit (Solarbio, Beijing, China). For IHC staining, the sections were permeabilized with 0.1% Triton X-100 for 10 min and blocked in 3% BSA (Sigma-Aldrich) in PBS for 30 min at room temperature. The sections were then incubated with the primary antibody against OCN (1:500, Abcam) and collagen I (COL1, 1:400, Abcam) at 4 °C overnight.

### Statistical analysis

Quantitative data were presented as means ± standard deviation (SD), and data were analyzed in SPSS 16.0 software. Shapiro-Wilk test and Kolmogorov-Smirnov test were used to test distribution of data. Unpaired *t* test was used to assess the statistical significance between two groups and one-way ANOVA was utilized in three or more groups. *P* value < 0.05 was considered as statistical significance.

## Results

### Osteogenic differentiation of hDPSCs

**Fig. 1:**
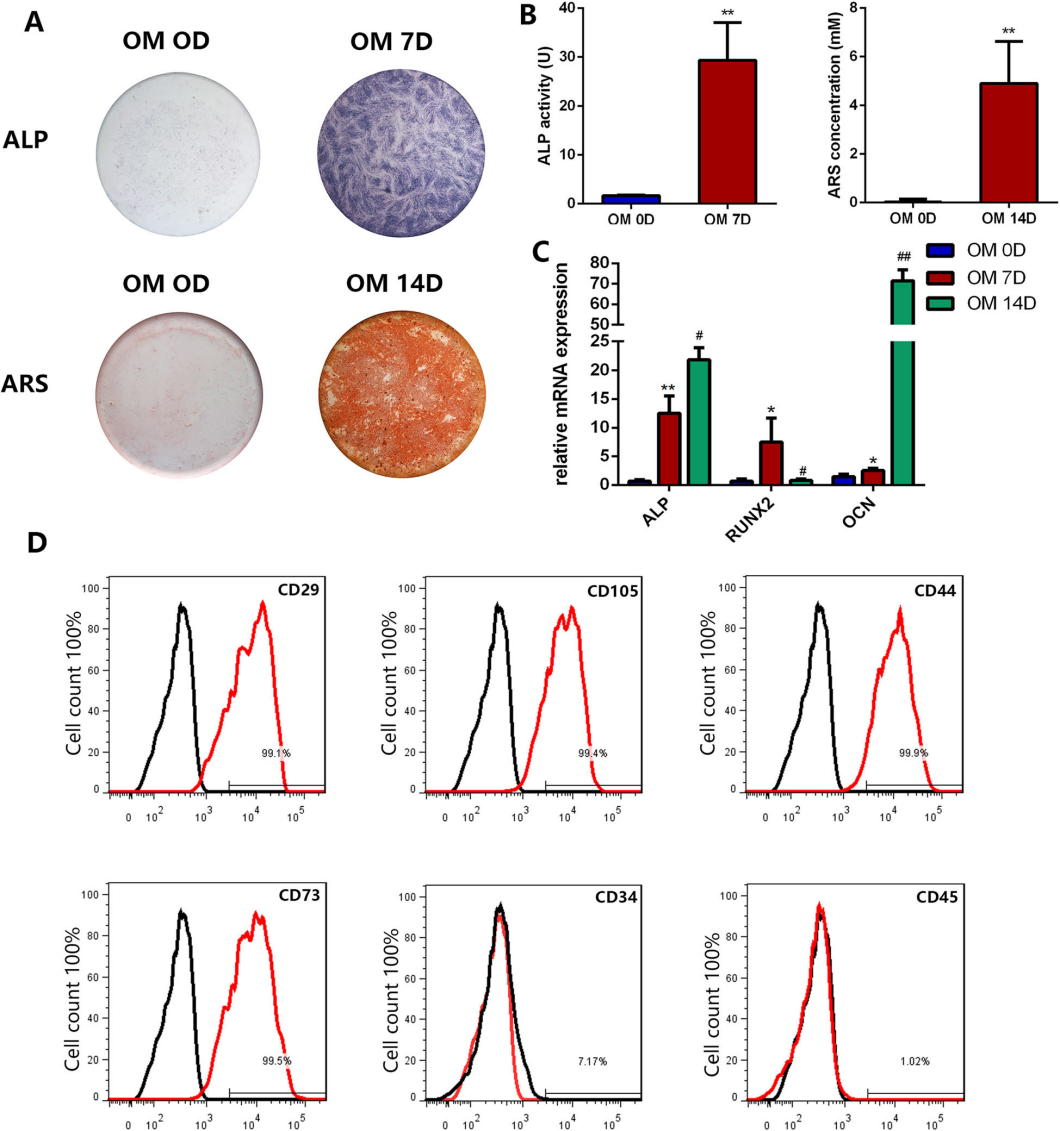
Osteogenesis ability and mesenchymal stem cell (MSC) property of human dental pulp stromal cells (hDPSCs). **a** Alkaline phosphatase (ALP) staining after 7 days’ osteogenic induction (OM 7D) and Alizarin Red S (ARS) staining after 14 days’ osteogenic induction (OM 14D). **b** Quantification of ALP and ARS. **c** mRNA expression levels of ALP, RUNX2, and OCN during hDPSC osteogenesis. **d** hDPSCs were positive for MSC-related surface markers. *p < 0.05 and **p < 0.01 compared with OM 0D. Three biological replicates were collected in each group

First, we confirmed the osteogenesis ability of hDPSCs. The intensity of ALP staining was significantly increased after 7 days (OM 7D), and calcified nodules of ARS staining were apparently spotted after osteogenic induction for 14 days (OM 14D) (Fig. 1a, b). Meanwhile, the mRNA expression levels of ALP, RUNX2, and OCN were significantly upregulated in the group of OM 7D and OM 14D compared to OM 0D (Fig. 1c). Flow cytometry showed high expression of MSC-associated surface markers CD 29, CD105, CD44, and CD73 and low expression of CD34 and CD45 (Fig. 1d).

### Differentially expressed circRNAs during osteogenic differentiation of hDPSCs

**Fig. 2:**
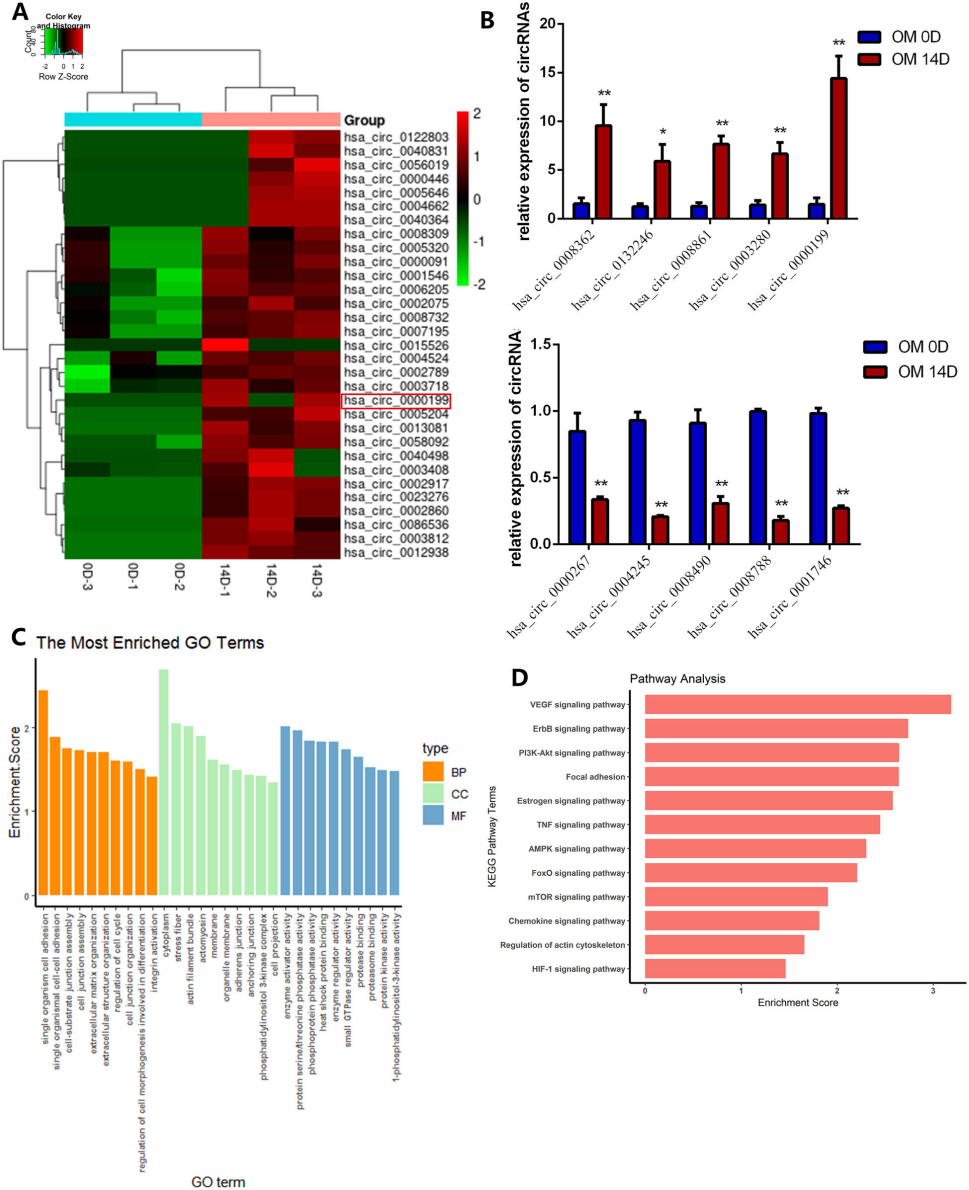
Microarray profiling showing differentially-expressed circRNAs during hDPSC osteogenesis. **a** The heatmap of the overall expression profiles of circRNAs during hDPSC osteogenesis for 0D and 14D (OM 0D vs OM 14D). **b** qRT-PCR of ten circRNAs for the validation of circRNA microarray results. **c** The top significant Gene Ontology (GO) terms of genes upregulated in OM 14D than OM 0D. **d** Kyoto Encyclopedia of Genes and Genomes (KEGG) pathway analysis of parental mRNAs showed the top 12 mRNA-enriched pathways. *p < 0.05 and **p < 0.01 compared with OM 0D. hDPSCs were harvested in two groups with three biological replicates

We identified circRNAs participating in osteogenesis of hDPSCs by circRNA array. The hierarchical clustering presented differentially expressed circRNAs in the OM 0D and OM 14D group (Fig. 2a). The volcano and scatter plots revealed the variation of circRNA expression between the two groups (Figure S1). We identified a total of 5846 circRNAs according to the RNA-seq analysis. There were 86 circRNAs expressing differentially with significance (fold change > 2.0; *p* < 0.05), among which 29 were downregulated while 57 upregulated in OM 14D compared with OM 0D.

To validate the RNA-Seq result, we selected ten differentially expressed circRNAs for qRT-PCR experiments. The characteristics of these circRNAs were listed in Tab S3. The expression levels of the ten selected circRNAs were consistent with the RNA-seq results (Fig. 2b). Among the ten circRNAs, circAKT3 was the most significantly upregulated, so we selected it for further research.

### Functional analysis of differentially expressed circRNAs

**Fig. 3:**
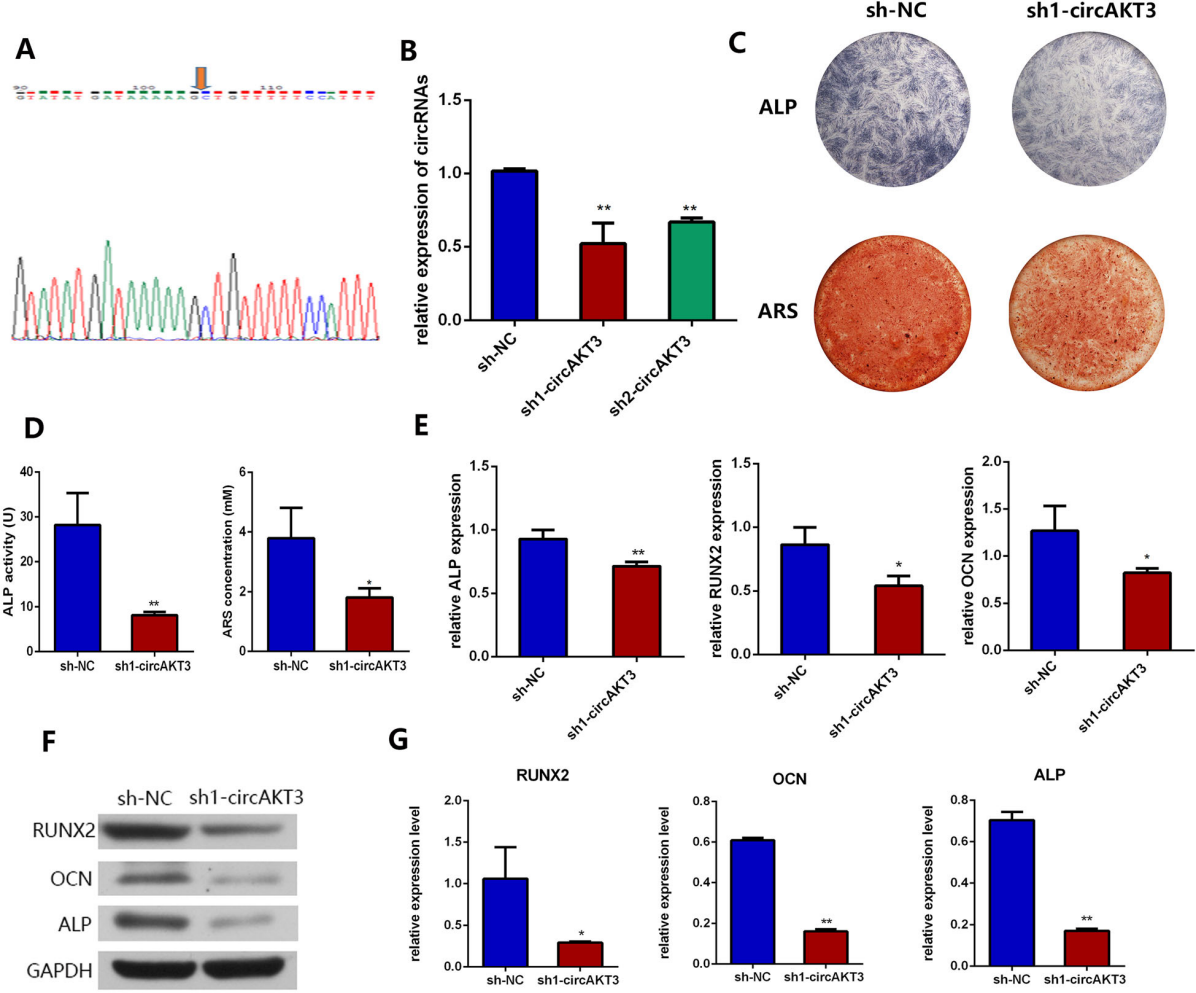
Knockdown of circAKT3 inhibited osteogenic differentiation of hDPSCs. **a** Sanger sequencing proved the circular property of circAKT3. The orange arrow shows the back-splicing site. **b** Transfection of sh1-circAKT3 or sh2-circAKT3 achieved knockdown of circAKT3 in hDPSCs as qRT-PCR suggested. **c** ALP and ARS staining of hDPSCs transfected by sh-NC and sh1-circAKT3. **d** Quantification of ALP and ARS staining. **e** Relative mRNA expression of ALP, RUNX2, and OCN was detected by qRT-PCR after osteogenic induction for 7 days. **f** Protein expression of RUNX2, OCN, ALP, and GAPDH after osteogenic induction detected by Western blot analysis. **g** Gray values of bands of RUNX2, OCN, ALP, and GAPDH *p < 0.05 and **p < 0.01 compared to the group of sh-NC. Three biological replicates were collected in each group

We performed GO and KEGG pathway analysis to analyze the parental genes of differentially expressed circRNAs (Fig. 2c). Some BP terms associated with osteogenesis processes were enriched, such as the single organism cell adhesion (GO:0098602), the single organismal cell-cell adhesion (GO:0016337), and regulation of cell morphogenesis involved in differentiation (GO:0010769) (Fig. 2c). In terms of CC, enriched terms included cytoplasm (GO:0005737), stress fibers (GO:0001725), actin filament bundle (GO:0032432), adherence junction (GO:0005912), and anchoring junction (GO:0070161) (Fig. 3a). As for MF, the most enriched terms include enzyme activator activity (GO:0008047), protein serine/threonine phosphatase activity (GO:0004722), and phosphoprotein phosphatase activity (GO:0004721) (Fig. 2c).

The KEGG pathway analysis identified 55 significant pathways related to the parental genes of differentially expressed circRNAs (Fig. 2d). Among them, 53 pathways were associated with upregulated circRNAs and two pathways were related with downregulated circRNAs. During osteogenic differentiation, host genes of differentially expressed circRNAs were functionally involved in VEGF signaling pathway (hsa04370), PI3K-Akt signaling pathway (hsa04151), focal adhesion (hsa04510), TNF signaling pathway (hsa04668), AMPK signaling pathway (hsa04152), and mTOR signaling pathways (hsa04150) (Fig. 2d).

### Knockdown of circAKT3 inhibits osteogenic differentiation of hDPSCs in vitro

Next, we examined the circular characteristic of circAKT3 by Sanger sequencing, confirming the specific splicing sites of circAKT3 (Fig. 3a).

To investigate the role of circAKT3 in hDPSC osteogenic differentiation, we transfected sh1-circAKT3 or sh2-circAKT3 to achieve the knockdown of circAKT3. Both sh1-circAKT3 and sh2-circAKT3 could effectively knockdown circAKT3 (Fig. 3b). We chose sh1-circAKT3 for further loss-of-function experiments. The ALP and ARS staining displayed that knockdown of circAKT3 significantly retarded osteogenic differentiation of hDPSCs (Fig. 3c, d). Meanwhile, knockdown of circAKT3 significantly reduced the expression levels of ALP, RUNX2, and OCN (Fig. 3e), suggesting its critical role in osteogenic differentiation. The protein levels of RUNX2 and OCN were examined for further confirmation. DPSCs in the sh1-circAKT3 group exhibited lower RUNX2, OCN, and ALP protein levels than those in the sh-NC group (Fig. 3f, g).

### circAKT3 directly targets miR-206

**Fig. 4:**
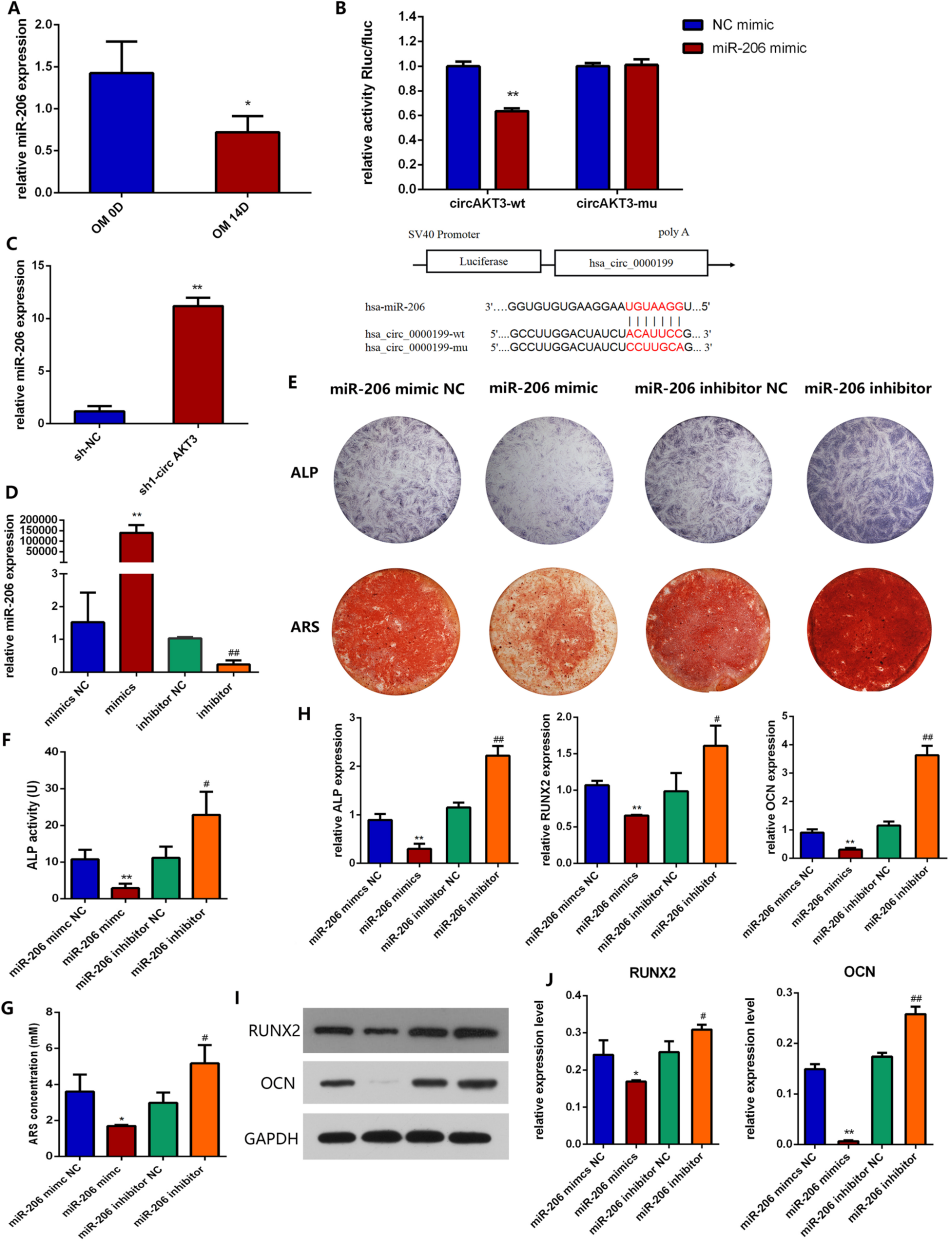
miR-206 inhibited osteogenic differentiation of hDPSCs. **a** Relative miR-206 expression during hDPSC osteogenesis. **b** Dual-luciferase reporter assay in 293 T cells validated the direct binding between circAKT3 and miR-206. **c** miR-206 expression in sh-NC and sh1-circAKT3 groups. **d** The efficiency of transient transduction of miR-206 mimics and miR-206 inhibitor. **e** ALP and ARS staining of hDPSCs transfected with miR-206 mimics and miR-206 inhibitor. **f-g** Quantification of ALP and ARS staining. **h** Relative mRNA expression of ALP, RUNX2, and OCN in hDPSCs transfected with miR-206 mimics and miR-206 inhibitor after osteogenic induction for 7 days. **i** Protein expression RUNX2, OCN, and GAPDH detected by Western blot analysis. **j** Gray values of bands of RUNX2, OCN, and GAPDH *p < 0.05 and **p < 0.01 compared to miR-206 mimic NC; ^#^*p* < 0.05 and ^##^*p* < 0.01 compared to miR-206 inhibitor NC. Three biological replicates were collected in each group

circRNAs can act as miRNA sponges to induce activation of downstream signaling. Therefore, we predicted candidate miRNAs according to miRanda and TargetScan Human 7.2 database to investigate whether circAKT3 could function in hDPSCs by sponging miRNAs. There is one predicted miRNA-binding site for miR-206 in circAKT3. During osteogenesis, the expression of circAKT3 was significantly enhanced while the expression of miR-206 was reduced (Fig. 4a). We then performed the luciferase activity assay to validate the prediction. circAKT3-wt reporter was strongly reduced by miR-206 mimics, while the circAKT3-mut reporter was not affected by miR-206 mimics (Fig. 4b), which indicated that miR-206 is a direct target of circAKT3. Moreover, qRT-PCR showed that the knockdown of circAKT3 by sh1-circAKT3 significantly upregulated the expression of miR-206 (Fig. 4c).

Then, we transfected hDPSCs with miR-206 mimic or inhibitor to investigate whether miR-206 could inhibit osteogenic differentiation of hDPSCs. The expression of miR-206 was significantly increased in the mimic group and decreased in the inhibitor group with significance, confirming the efficiency of the transfection (Fig. 4d). The intensity of ALP and ARS staining was reduced by miR-206 mimic and enhanced by miR-206 inhibitor (Fig. 4e-g). Moreover, qRT-PCR showed the miR-206 mimic significantly suppressed the expression of ALP, RUNX2, and OCN, while miR-206 inhibitor significantly enhanced the expression of the osteogenic genes (Fig. 4h). Protein expression of RUNX2 and OCN was decreased by miR-206 mimic and increased by miR-206 inhibitor (Fig. 4i, j).

### Dynamic effects of circAKT3 and miR-206 on osteogenic differentiation of hDPSCs

**Fig. 5:**
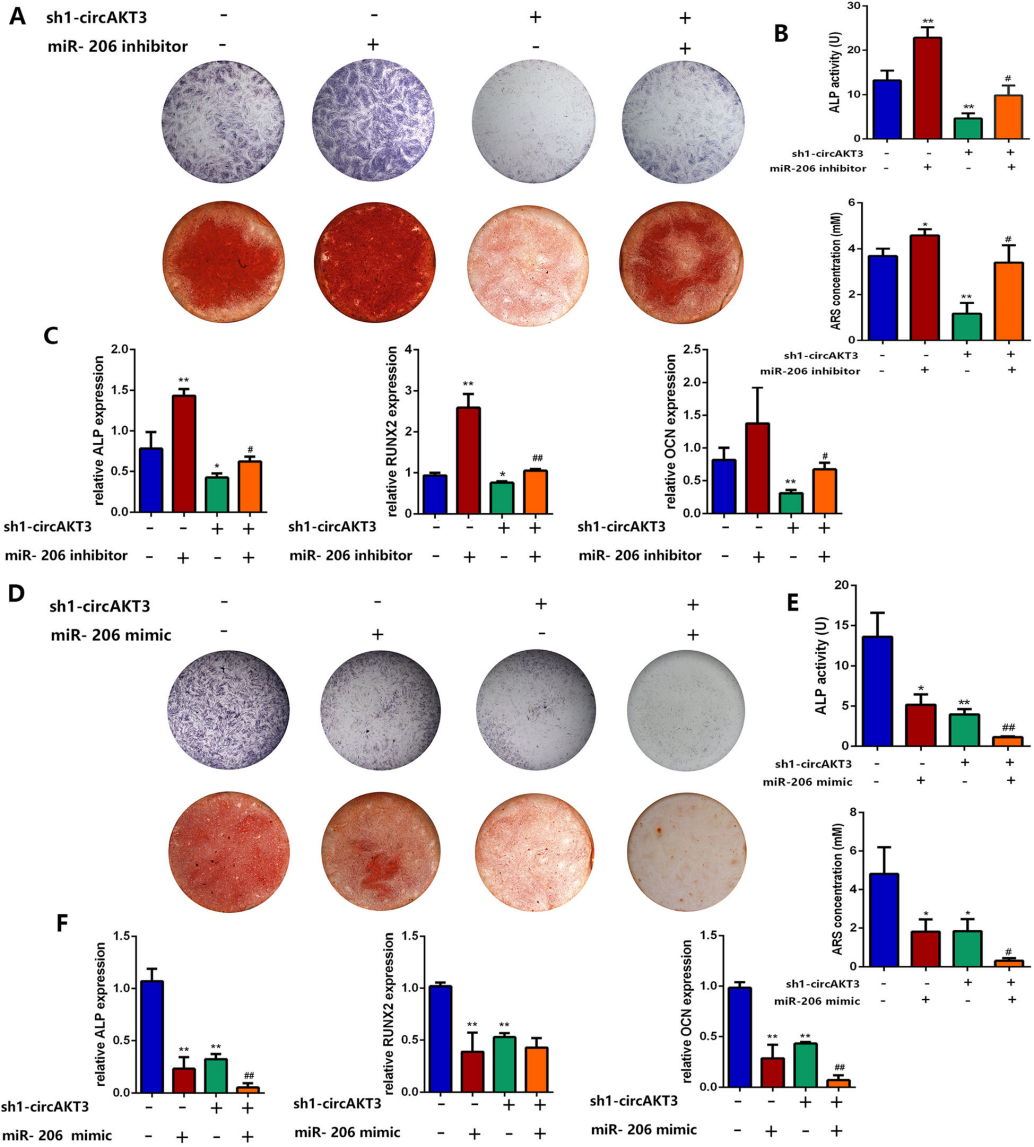
Dynamic effects of circAKT3 and miR-206 on hDPSC osteogenesis. **a**–**c** ALP and ARS staining, quantification, and mRNA expression of hDPSCs co-transfected with sh1-circAKT3 and miR-206 inhibitor. **d**–**f** ALP and ARS staining, quantification, and mRNA expression of hDPSC co-transfected with sh1-circAKT3 and miR-206 mimic. *p < 0.05 and **p < 0.01 compared to sh-NC + miR-206 mimic/inhibitor NC; ^#^*p* < 0.05 and ^##^*p* < 0.01 compared to sh1-circAKT3 + miR-206 mimic/inhibitor NC. Three biological replicates were collected in each group

To investigate the dynamic effects of circAKT3 and miR-206 on hDPSC osteogenesis, we co-transfected hDPSCs with sh1-circAKT3 and miR-206 mimic or inhibitor. miR-206 inhibitor partially reversed the suppressive effect of sh1-circAKT3 on ALP/ARS staining and mRNA expression of ALP, RUNX2, and OCN (Fig. 5a–c), while miR-mimic aggravated the inhibitory effect of sh1-circAKT3 (Fig. 5d–f).

**Fig. 6:**
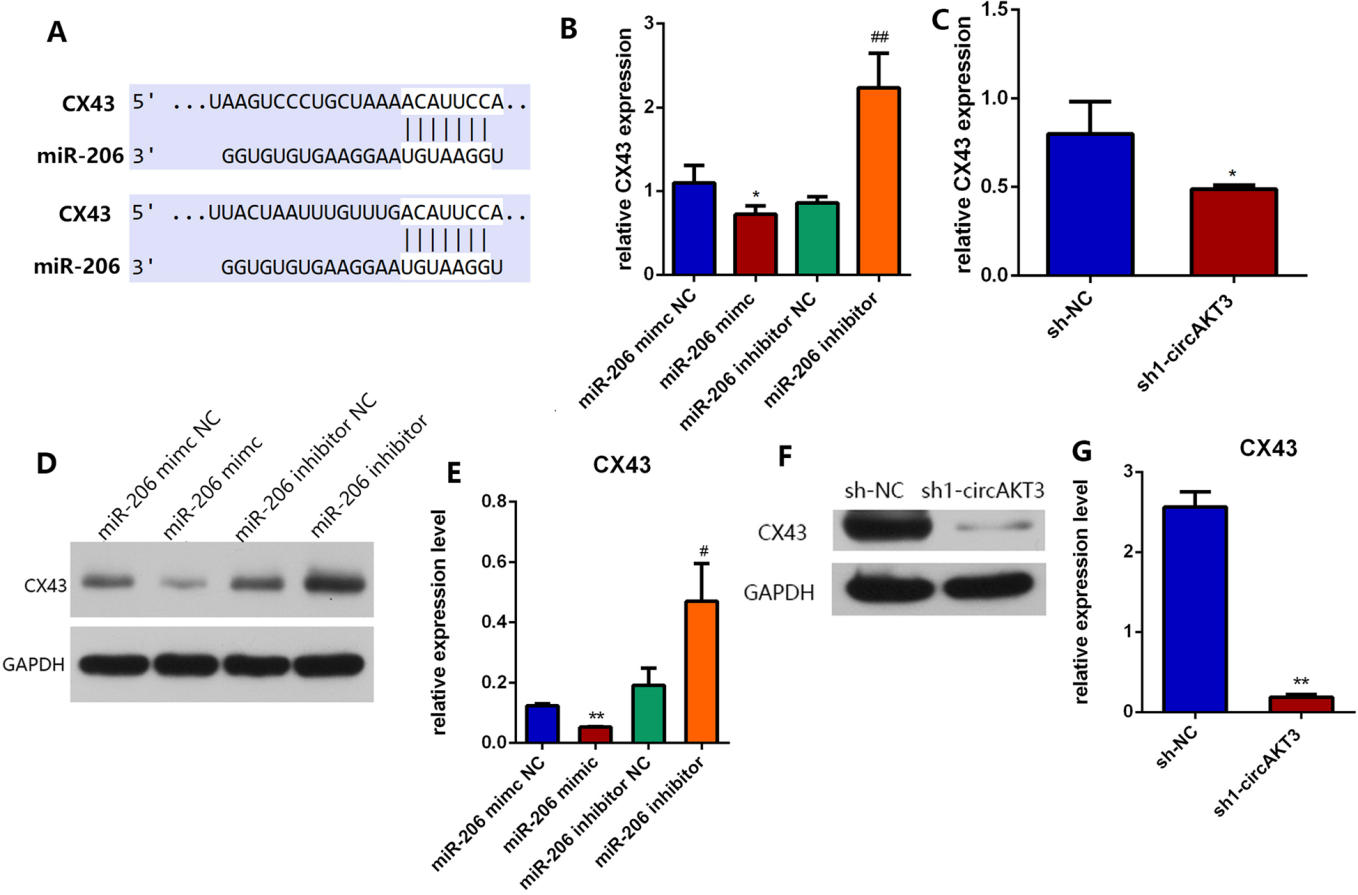
Connexin 43 (CX 43) was a potential target of circAKT3/miR-206. **a** The predicted binding sites of miR-206 and the 3′ untranslated region (UTR) of CX43. **b** CX43 expression measured by qRT-PCR in miR-206 mimic and inhibitor groups. **c** CX43 mRNA expression in circAKT3 knockdown group. **d**, **e** Protein levels and gray values of CX43 measured by Western blot in miR-206 mimic and inhibitor groups. **f**, **g** Protein levels and gray values of CX43 in circAKT3 knockdown group. *p < 0.05 and **p < 0.01 compared to sh-NC or miR-206 mimic NC; ^#^*p* < 0.05 and ^##^*p* < 0.01 compared miR-206 inhibitor NC. Three biological replicates were collected in each group

To further investigate the downstream molecular mechanism of circAKT3/miR-206 regulating the osteogenic differentiation of hDPSCs, we search potential targets of miR-206 in two algorithms (miRanda and TargetScan Human 7.2). Notably, we identified that the 3′ untranslated region (UTR) of CX 43 contained miR-206 binding sites (Fig. 6a). Some previous studies have verified the direct binding of CX 43 mRNA and miR-206 by luciferase reporter assay [22, 23]. To validate whether CX 43 acts as the target of miR-206, we detected mRNA and protein levels of CX43 in hDPSCs transfected with miR-206 mimic/inhibitor and sh1-circAKT3, respectively. miR-206 mimic significantly decreased the expression of CX43 while miR-206 inhibitor enhanced CX 43 expression (Fig. 6b, d). Knockdown of circAKT3 decreased mRNA and protein levels of CX43 (Fig. 6c, f).

### In vivo effects of circAKT3 on an osteogenesis mice model

**Fig. 7:**
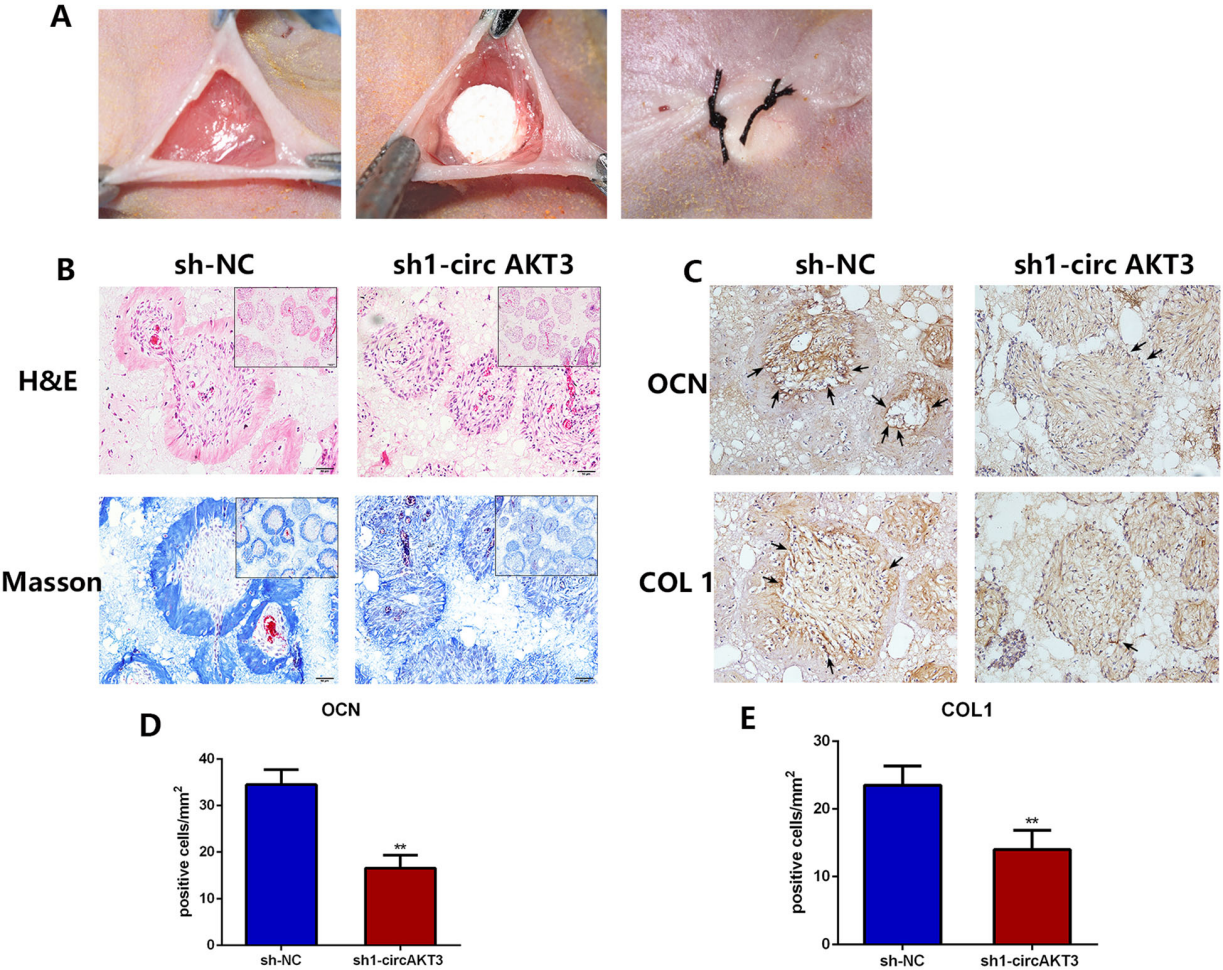
Knockdown of circAKT3 inhibited hDPSC mineralization in vivo. **a** Transplantation surgery of β-TCP/hDPCs composite on nude mice. **b** Hematoxylin and eosin (H&E) staining and Masson’s trichrome staining of β-TCP/hDPCs composites after 2 months. **c**–**e** Immunohistochemistry staining and quantification of OCN and COL1 expression of transplantation composites. Black arrow points to positive cells. Four biological replicates were collected in each group

**Fig. 8:**
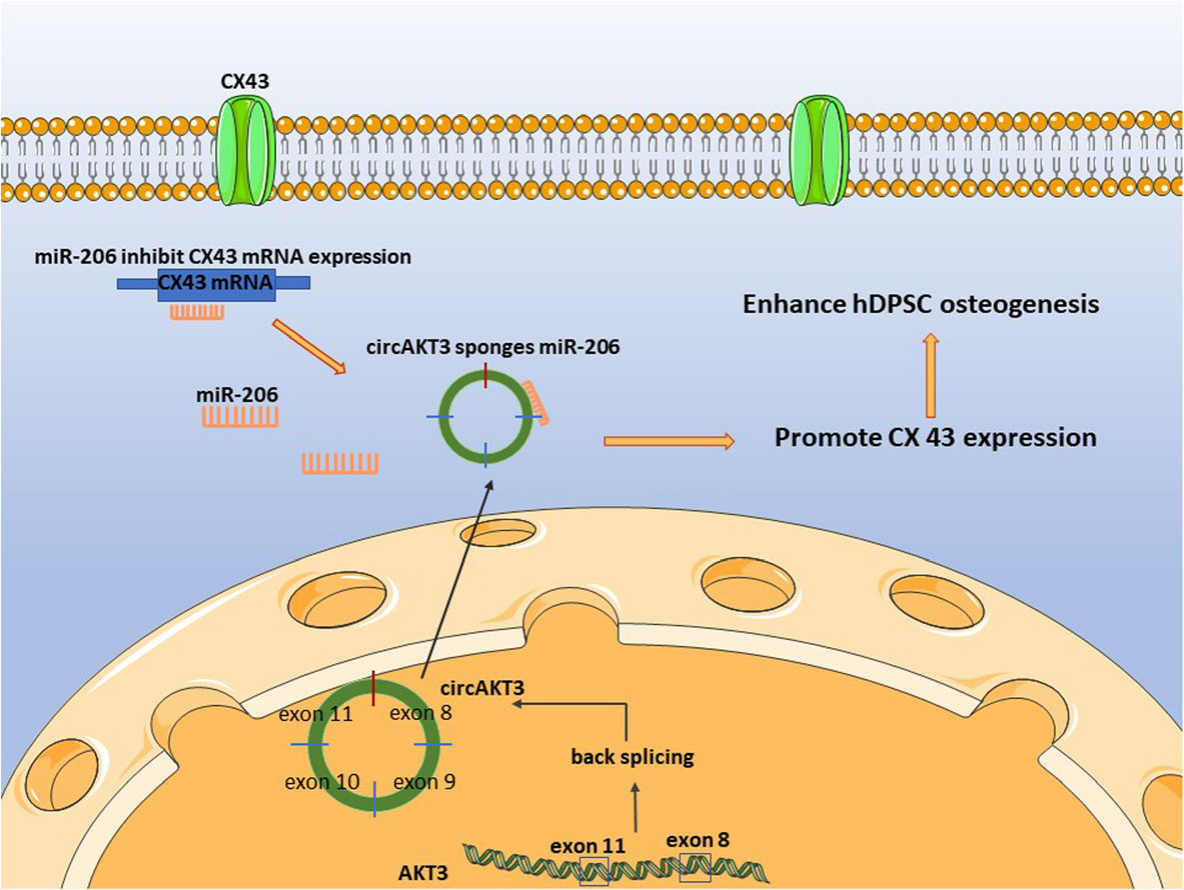
circAKT3 targets miR-206/CX43 in hDPSC osteogenesis

We conducted subcutaneous transplantation of β-TCP/ hDPSC composites in BALB/c nude mice to further explore the role of circAKT3 in hDPSC mineralization in vivo (Fig. 7a). Before loaded on β-TCP, the cells were transfected with sh-NC or sh1-circAKT3. After 8 weeks, H&E and Masson’s trichrome staining showed more mineralized nodules in the sh-NC group while dispersed blue-stained collagen fibers in the group of sh1-circAKT3 (Fig. 7b). Additionally, we examined the expression of osteogenic genes through IHC staining. Consistently, the expression of OCN and COL1 was remarkably suppressed by sh1-circAKT3 (Fig. 7c–e). Our results indicated that circAKT3 served as a positive modulator during osteogenic differentiation (Fig. 8).

## Discussion

Herein, we performed microarray profiling analyses on osteogenic-induced hDPSCs and identified that circAKT3 was significantly upregulated during osteogenesis. The knockdown of circAKT3 significantly suppressed osteogenesis in vitro. Then, circAKT3 was predicted and further validated to directly bind to miR-206, which could target CX43 mRNA. In the in vivo bone formation model, silencing circAKT3 remarkably inhibited subcutaneous mineralization. Therefore, circAKT3 modulated hDPSC osteogenesis possibly through abolishing the potential suppressive role of miR-206 on CX43.

Sponging miRNAs is one of the most important and widely-reported functional mechanisms of circRNAs [24, 25]. Highly abundant circRNAs contain binding sites of miRNAs, competently binding to miRNAs, and suppress the inhibitive effect of miRNAs on their target genes. There is increasing evidence of the involvement of circRNAs in the osteogenesis of stem cells by sponging miRNAs. For instance, Li et al. have found that circCDR1 could enhance osteogenesis of periodontal ligament stem cells through modulating miR-7/GDF5/SMAD axis [26]. During osteogenesis of maxillary sinus membrane stem cells, Peng et al. reported that circRNA_33287 acted as miR-214-3p sponge, alleviating the inhibitory effect of miR-214-3p on Runx 3 [27]. Moreover, our previous studies suggested that circPOMT1 and circMCM3AP inhibited osteogenesis of hASCs by targeting miR-6881-3p [20], circRFWD2 and circINO80 played a positive role in NELL-1-induced osteogenesis of hASCs via sponging hsa-miR-6817-5p [28]. Although the above studies substantiated the engagement of circRNAs in osteogenic differentiation, the effects of circRNAs on the osteogenesis of hDPSCs and underlying mechanisms have not been fully elucidated.

hDPSCs have drawn increasing attention as potential sources of MSCs for regenerative medicine due to its merits in high proliferation rate, plasticity in multi-lineage differentiation, and convenience of acquirement. Dental MSCs origin from neural crest cells and a latest lineage tracing study confirmed that dental MSCs derived from a population of peripheral nerve-associated glias [29]. The origin of hDPSCs gives them unique neurovascular properties, which make them a promising population for tissue regeneration. Indeed, numerous studies described that hDPSCs could differentiate into osteoblasts, odontoblasts, chondrocytes, adipocytes, and neural-like cells [30–32].

circAKT3 was firstly reported to function in cisplatin resistance in gastric cancer [33]. Huang et al. found that circAKT3 could sponge miR-198 and abolish the inhibitive effect of miR-198 on its target gene PIK3R1, which activated PI3K/AKT signaling pathway [33]. In their study, the resistance of circAKT3 to RNase R treatment was proven, the head-to-tail splicing site was verified, and the possibility of trans-splicing/genome recombination was excluded [33].

Based on bioinformatic analysis and luciferase activity assay, we chose miR-206 as the sponging target of circAKT3 in the regulation of osteogenesis. miR-206 has been found to inhibit osteogenesis in osteoblasts and MSCs [22, 34]. Inose et al. firstly observed gradual decreasing level of miR-206 during the course of osteoblast differentiation in mouse osteoblasts [22]. They found overexpression of miR-206 inhibited osteoblast differentiation by targeting 3′UTR region of Cx43 mRNA and downregulating Cx43 protein expression [22]. The direct binding of miR-206 and 3′UTR region CX43 mRNA in human cells was also confirmed by several previous studies [35, 36].

Connexin 43 (CX43), also known as gap junction protein alpha 1 (GJA1) in human, expresses in multiple cell types and facilitates inter-cellular communication through the docking of gap junctions between two cells, or the formation of unpaired hemichannels [37]. CX 43 plays a critical role in osteogenesis or odontogenesis. CX43-deficient mice displayed low bone mass and delayed ossification due to osteoblast dysfunction [38, 39]. Inhibition of CX43 could impair osteogenesis of BMSCs, indicating its central role throughout the osteogenic process [40]. Moreover, CX43 overexpression could amplify odontogenesis of hDPSCs through Erk1/2, while inhibition of CX43 expression suppressed odontogenesis [41]. Chung et al. also found that blocking CX43 by antisense oligonucleotides could inhibit odontogenic differentiation of rat dental pulp cells [42].

The investigations on biological implications of circRNAs in cell behaviors are still in a preliminary stage. Apart from acting as miRNA sponges, the modes of actions of circRNAs also include directly interacting with proteins, encoding proteins, and affecting transcription or splicing of their parental genes [43]. Our study focused on the mechanism of sponging miRNAs. Future experiments including RNA-pull down assay, RNA immunoprecipitation, and fluorescence in situ hybridization are still needed to test other possibilities of circRNA actions in osteogenic differentiation of hDPSCs [44].

Understanding the contribution of circRNAs to the osteogenesis is still in its infancy. Although RNA-seq has identified a great amount of putative circRNAs in osteogenesis of stem cells, the majority of them are still waiting for further verification and functional investigation. Moreover, the advent of some state-of-the-art techniques, like single-cell RNA-seq and Digital Spatial Profiling, is likely to drive the further development of circRNA identification and functional characterization [45]. Additionally, functions of circRNAs in various biological processes and diseases have brought the application of circRNA molecules as gene therapies into focus. Therefore, future studies are anticipated to explore the functions of circRNAs in osteogenesis and the possibility of their utilization in bone regeneration.

## Conclusion

In conclusion, our study was the first to demonstrate the function of circAKT3 in osteogenic differentiation of hDPSCs, revealing the mechanism that circAKT3 could sponge miR-206 to affect the expression of CX43 in hDPCSs. Our results indicated that circAKT3 might be a novel therapeutic target for bone tissue regeneration by hDPSCs.

## Supplementary Information

**Additional file 1: Figure S1.** A. Volcano plot representing differentially-expressed circRNAs between osteogenic induction for 0D and 14D (OM 0D vs OM 14D). Red dots on the left side represented significantly downregulated genes (Fold change≥2; p≤0.05) in OM 14D than OM 0D, while those on the right side represented significantly upregulated genes (Fold change≥2; p≤0.05). Grey dots represented differentially expressed genes with p value >0.05. C. Scatter plot diagram showing the expression correlation of these circRNAs. The red dots on the left side stood for the downregulated circRNAs and the green dots on the right side stood for the upregulated circRNAs with significant differences (p<0.05), while the purple dots stood for the circRNAs expressing differentially without significance. **Table S1.** Sequences of RNA oligoribonucleotide. **Table S2.** Primers for quantitative expression analysis of qRT-PCR. **Table S3.** The list of circRNA for qRT-PCR validation.

## Data Availability

The datasets used during the current study are available from the corresponding authors on reasonable request.

## Abbreviations

ALP: Alkaline phosphatase
ARS: Alizarin Red S
BMSCs: Bone marrow mesenchymal stromal cells
BP: Biological processes
CC: Cellular components
circRNAs: Circular RNAs
CX43: Connexin 43
EDTA: Ethylenediaminetetraacetic acid
GAPDH: Glyceraldehyde 3-phosphate dehydrogenase
GJA1: Gap junction protein alpha 1
GO: Gene Ontology
H&E: Hematoxylin and eosin
hDPSCs: Human dental pulp stromal cells
IHC: Immunohistochemistry
KEGG: Kyoto Encyclopedia of Genes and Genomes
MF: Molecular functions
miRNA: MicroRNA
MSCs: Mesenchymal stromal cells
OCN: Osteocalcin
OM: Osteogenic-induction medium
PVDF: Polyvinylidene difluoride
qRT-PCR: Quantitative reverse transcription polymerase chain reaction
RBPs: RNA-binding proteins
RIPA: Radioimmunoprecipitation assay
RNA-seq: RNA sequencing
RUNX2: Runt-related transcription factor 2
SD: Standard deviation
SDS-PAGE: Sulfate-polyacrylamide gel electrophoresis
UTR: Untranslated region
β-TCP: Beta-tricalcium phosphate
