# Supplementary material for: circAKT3 positively regulates osteogenic differentiation of human dental pulp stromal cells via miR-206/CX43 axis
Source: Stem Cell Res Ther. 2020 Dec 9;11:531. doi: 10.1186/s13287-020-02058-y (PMC7726914; doi:10.1186/s13287-020-02058-y)
Supplement: Supplementary file 1 — Additional file 1: Figure S1. A. Volcano plot representing differentially-expressed circRNAs between osteogenic induction for 0D and 14D (OM 0D vs OM 14D). Red dots on the left side represented significantly downregulated genes (Fold change≥2; p≤0.05) in OM 14D than OM 0D, while those on the right side represented significantly upregulated genes (Fold change≥2; p≤0.05). Grey dots represented differentially expressed genes with p value >0.05. C. Scatter plot diagram showing the expression correlation of these circRNAs. The red dots on the left side stood for the downregulated circRNAs and the green dots on the right side stood for the upregulated circRNAs with significant differences (p<0.05), while the purple dots stood for the circRNAs expressing differentially without significance. Table S1. Sequences of RNA oligoribonucleotide. Table S2. Primers for quantitative expression analysis of qRT-PCR. Table S3. The list of circRNA for qRT-PCR validation. [file 13287_2020_2058_MOESM1_ESM.pdf]

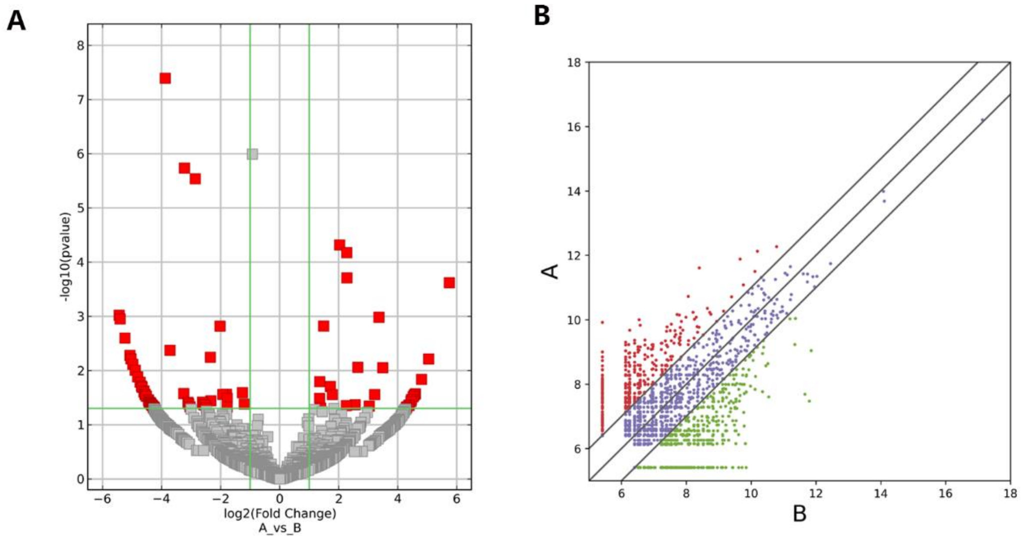

Figure S1. A. Volcano plot representing differentially-expressed circRNAs between osteogenic induction for 0D and 14D (OM 0D vs OM 14D). Red dots on the left side represented significantly downregulated genes (Fold change≥2; p≤0.05) in OM 14D than OM 0D, while those on the right side represented significantly upregulated genes (Fold change≥2; p≤0.05). Grey dots represented differentially expressed genes with p value >0.05. C. Scatter plot diagram showing the expression correlation of these circRNAs. The red dots on the left side stood for the downregulated circRNAs and the green dots on the right side stood for the upregulated circRNAs with significant differences ($p < 0.05$), while the purple dots stood for the circRNAs expressing differentially without significance.

Table S1 Sequences of RNA oligoribonucleotide

Gene name	Sequences
sh1-circAKT3	AUGGAAAAACAGCUUUUUAUC
sh2-circAKT3	UGGAAAAACAGCUUUUUAUCA
miR-206 mimic NC	UUGUACUACACAAAAGUACUG
miR-206 mimic	UGGAAUGUAAGGAAGUGUGUGG
miR-206 inhibitor NC	CAGUACUUUUGUGUAGUACAA
miR-206 inhibitor	CCACACACUUCCUUACAUUCCA

Table S2 Primers for quantitative expression analysis of qRT-PCR

Gene name	Primer Sequence	
circAKT3	Forward	ATCATTGCTTTCAGGGCTCT
	Reverse	CACCCGCTCTCTCGACAAAT
miR-206	Forward	GGGTGGAATGTAAGGAAGT
	Reverse	CAGTGCGTGTCGTGGAGT
ALP	Forward	CTATCCTGGCTCCGTGCTCC
	Reverse	GTTAACTGATGTTCCAATCCTGCG
RUNX2	Forward	CCTTTACTTACACCCCGCCA
	Reverse	GGATCCTGACGAAGTGCCAT
OCN	Forward	ATTGTGGCTCACCCTCCATC
	Reverse	CCAGCCTCCAGCACTGTTTA
CX43	Forward	ACCATCTCTAACTCCCATGCACAG
	Reverse	TGAAGGTCGCTGGTCCACAAT
hsa_circ_0003280	Forward	TCCGAGTCAGATTGGAGCAT
	Reverse	TCGCTCACCATCTGCTGTAA
hsa_circ_0008861	Forward	TGAAGTTAAGCGCCTCCTTG
	Reverse	GCATTCCACACAACTACAATTCC
hsa_circ_0132246	Forward	CCAGCCTTAGTGCCAAAGTG
	Reverse	TCTGTCTTGTTCGCTCCTCC
hsa_circ_0008362	Forward	CCGAGTCTGGTCCATTGCTA
	Reverse	TTCAGCAGAATCCCCTCTCG
hsa_circ_0001746	Forward	TGATGCTTGCGAAGAGTTGA
	Reverse	CTGAACTATAGCAGGCCTTTCG
hsa_circ_0008788	Forward	TGTACTCTTTGTGGCTCTGC
	Reverse	GCGTGGTGAGATGTTTCCAA
hsa_circ_0008490	Forward	GGAGGTGGTGTAAAGGAAGC
	Reverse	CGGTCCATCAGAATTGCCAG
hsa_circ_0004245	Forward	AATGTTGCTGCACCAAAGGA
	Reverse	ACTTTGTCTGGAGAGCTTGTG
hsa_circ_0000267	Forward	CCTTCTCTAGACCTTGCCAAGA
	Reverse	CTCCCGGTGCCATAGTGAG
GAPDH	Forward	GGAGCGAGATCCCTCCAAAAT
	Reverse	GGCTGTTGTCATACTTCTCATGG
U6	Forward	CTCGCTTCGGCAGCACA
	Reverse	AACGCTTCACGAATTTGCGT

Table S3 The list of circRNA for qRT-PCR validation

ID	logFC	p	txStart	txEnd
hsa_circ_0000199	4.311708	0.0443	178921331	178922376
hsa_circ_0003280	4.68196	0.01935	137904613	137906830
hsa_circ_0008861	4.812407	0.013034	76745584	76760634
hsa_circ_0132246	5.406812	0.001113	73005639	73043538
hsa_circ_0008362	5.435274	0.000955	103427642	103436193
hsa_circ_0001746	2.646399	0.008724	131060182	131084192
hsa_circ_0008788	3.217714	0.027623	215342541	215345526
hsa_circ_0008490	4.524877	0.030284	67546221	67571058
hsa_circ_0004245	4.558569	0.028123	61013821	61034674
hsa_circ_0000267	5.048809	0.006111	126370175	126370948
